# Supplementary material for: A network dynamical simulation model for the study of antibiotic resistance in microbial communities
Source: Gut Microbes. 2026 Sep 19;18(1):2734703. doi: 10.1080/19490976.2026.2734703 (PMC13596919; doi:10.1080/19490976.2026.2734703)
Supplement: Supplementary file 1 [file KGMI_A_2734703_SM9053.zip › supplementary_file_1/Figures/ba_delta_mean_distance_gc_n1000.pdf]

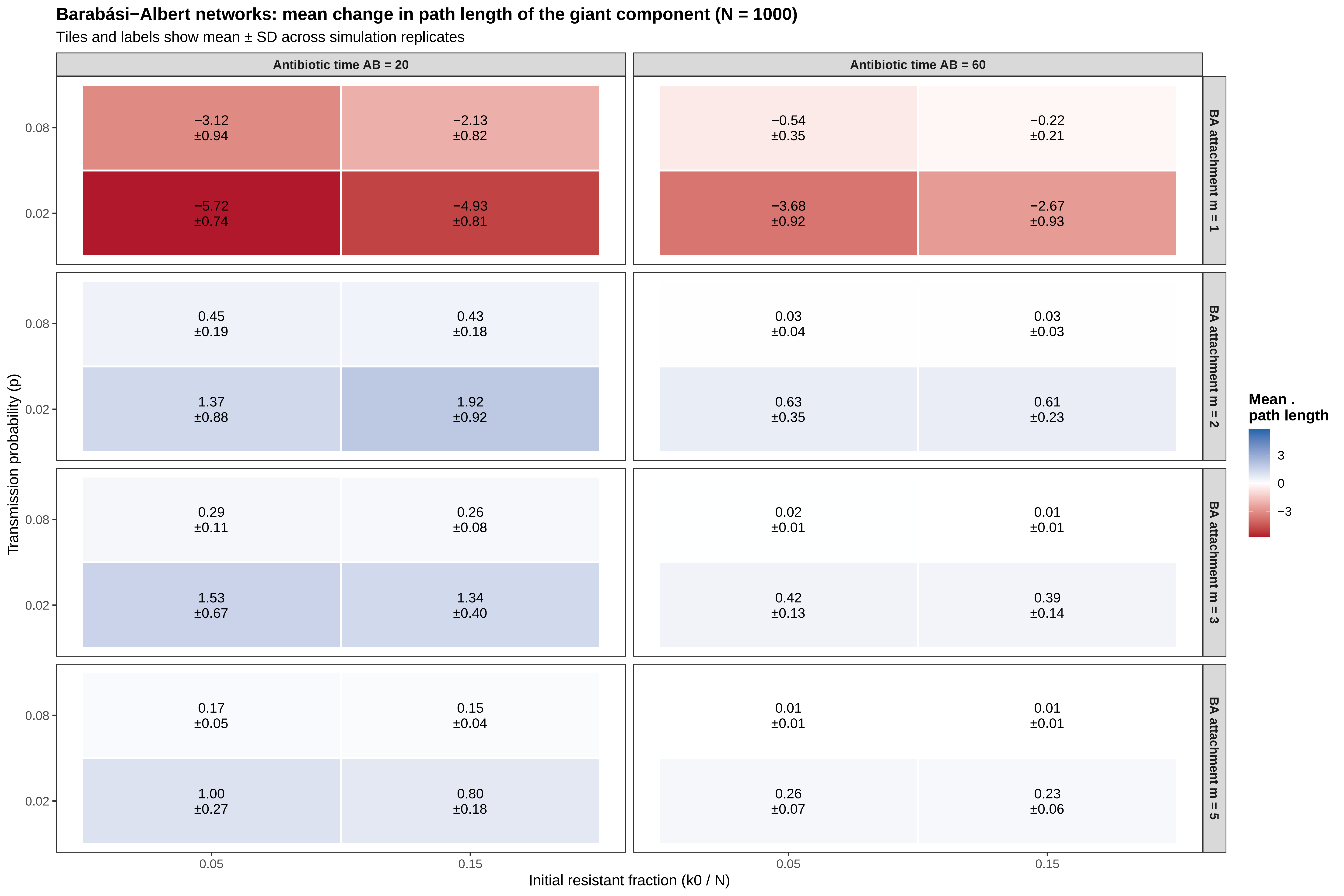
Barabási−Albert networks: mean change in path length of the giant component (N = 1000)
Tiles and labels show mean ± SD across simulation replicates
Antibiotic time AB = 20
Antibiotic time AB = 60
BA attachment m = 1
BA attachment m = 2
BA attachment m = 3
BA attachment m = 5
−3.12 ±0.94
−2.13 ±0.82
−5.72 ±0.74
−4.93 ±0.81
−0.54 ±0.35
−0.22 ±0.21
−3.68 ±0.92
−2.67 ±0.93
0.45 ±0.19
0.43 ±0.18
1.37 ±0.88
1.92 ±0.92
0.03 ±0.04
0.03 ±0.03
0.63 ±0.35
0.61 ±0.23
0.29 ±0.11
0.26 ±0.08
1.53 ±0.67
1.34 ±0.40
0.02 ±0.01
0.01 ±0.01
0.42 ±0.13
0.39 ±0.14
0.17 ±0.05
0.15 ±0.04
1.00 ±0.27
0.80 ±0.18
0.01 ±0.01
0.01 ±0.01
0.26 ±0.07
0.23 ±0.06
Transmission probability (p)
0.08
0.02
Initial resistant fraction (k0 / N)
0.05
0.15
Mean .
path length
3
0
−3
